# Determinants of patient satisfaction in continuous positive airway pressure therapy for obstructive sleep apnea: A multivariate analysis of knowledge, technical, and psychosocial factors

**DOI:** 10.1007/s11325-025-03438-5

**Published:** 2025-08-07

**Authors:** Marcel Braun, Sarah Dietz-Terjung, Torsten Eggert, Christoph Schoebel

**Affiliations:** 1 Department of Pneumology, West German Lung Center, University Medicine Essen – Ruhrlandklinik, University Duisburg-Essen, Duisburg, Germany; 2 Faculty of Sleep and Telemedicine, West German Lung Center, University Medicine Essen – Ruhrlandklinik, University Duisburg-Essen, Tueschener Weg 40, 45239 Duisburg, Essen, Germany

**Keywords:** Patient satisfaction, PAP adherence, Patient experience, Treatment predictors, Patient-reported outcomes

## Abstract

**Background:**

Positive airway pressure (PAP) therapy is the primary treatment for obstructive sleep apnea (OSA), but long-term adherence remains challenging. This study aimed to identify key predictors of patient satisfaction with PAP therapy by examining the interrelationships between disease knowledge, care provision, health beliefs, and patient-reported experiences.

**Methods:**

A cross-sectional study was conducted at a tertiary sleep center in Germany. Adult patients with confirmed OSA diagnosis and experience with PAP therapy completed a comprehensive questionnaire assessing four domains: health attitudes and self-management, OSA care provision perceptions, OSA disease knowledge, and treatment experience. Multiple regression analysis was performed to identify predictors of treatment satisfaction.

**Results:**

Among 148 participants (72.8% male, mean age 57.9 ± 11.8 years), 58.8% were active PAP users. High levels of family support (> 90%) and disease knowledge were reported, with 94.4% acknowledging the importance of regular treatment. Treatment experience emerged as the primary predictor of satisfaction in domain-level analysis. The final regression model identified four significant predictors explaining 75.9% of variance in satisfaction: treatment effectiveness (β = 0.304, *p* <. 001), routine integration (β = 0.286, *p* =. 001), nocturnal awakening (β = 0.273, *p* <. 001), and mask removal (β = 0.198, *p* =. 007). Patient satisfaction negatively correlated with alternative treatment usage (τ=-0.274, *p* <. 001) and positively with disease duration (τ = 0.187, *p* =. 008).

**Conclusion:**

PAP therapy satisfaction is predominantly influenced by treatment-specific factors rather than general health attitudes or care provision perceptions. Understanding these predictors may help clinicians identify patients at risk for low satisfaction and target interventions accordingly.

**Supplementary Information:**

The online version contains supplementary material available at 10.1007/s11325-025-03438-5.

## Introduction

Obstructive sleep apnea (OSA) is one of the most prevalent chronic sleep disorders, that impacts up to 936 million adults globally, with prevalence in the adult population ranging between 23 and 46% in developed countries [1]. OSA is characterized by repeated collapse of upper airway soft tissues during sleep, leading to disruptions in gas exchange and subsequent impact on sleep architecture due to respiration-related arousals. Recent research emphasizes that OSA pathophysiology involves a complex relationship of anatomical vulnerability, arousal threshold, muscle responsiveness, and ventilatory control stability [2, 3, 4, 5]. If left untreated, the condition can lead to significant health consequences, including increased risk of cardiovascular diseases, metabolic disorders, and neurological complications [6]. Current estimates suggest that in Germany, moderate to severe OSA requiring treatment affects up to 30% of men and 13% of women [7].

Positive airway pressure (PAP) therapy remains the primary treatment modality, demonstrating high efficacy in reducing respiratory events and improving patient symptoms. However, long-term adherence can be challenging, with studies reporting chronic adherence rates ranging from 30 to 60% across different patient populations. This variability highlights the complex nature of treatment engagement, which extends beyond clinical effectiveness [8, 9, 10].

Patient experience with OSA treatment is influenced by multiple elements, including individual knowledge, psychological responses, social support systems, and treatment-specific factors [11, 12]. While the current literature suggests that treatment experience, health beliefs, and perceptions of care provision significantly impact patient satisfaction and adherence, most studies have approached these factors in isolation. The objective of this study was to conduct a broad analysis of aspects influencing PAP therapy satisfaction by examining the interrelationships between these factors and patient-reported experiences. It addresses a gap by integrating different determinants of satisfaction into a unified model to examine how these domains interact over time through disease-stage stratification. Such insights may inform more personalized adherence strategies.

## Methods

### Study design and setting

This cross-sectional, mixed-methods observational study was conducted at a tertiary sleep medicine center in western Germany serving a broad catchment area of urban and rural populations. The majority of patients are referred by pulmonologists or general practitioners. While PAP therapy is the standard first-line treatment provided, alternative treatments like oral appliances and positional therapy are increasingly indicated. Surgical or neurostimulation-based treatments are managed in collaboration with local ENT specialists. No formal sample size calculation was conducted a priori, as this study was exploratory in nature and based on a convenience sample of consecutively treated patients. The research employed a comprehensive survey approach to evaluate and explore patient experiences with PAP therapy for the treatment of OSA. Participants were recruited from the outpatient clinic and included adults with a confirmed diagnosis of OSA who were currently using or had previously used PAP therapy and were able to provide informed consent and complete the survey. Exclusion criteria included severe cognitive impairment, inability to understand the questionnaire, or non-German language proficiency. No participants were excluded based on sleep-related comorbidities such as depression or insomnia, unless these impaired their ability to complete the questionnaire.

### Data collection instruments

A paper-format survey was employed to collect data on patient experiences and administered after obtaining informed consent. Additional information was collected from case records.

The survey instrument consisted of 38 items across four domains, which were included for validation of the questionnaire:

- Health attitudes and self-management (7 items).
- OSA care provision perceptions (12 items).
- OSA disease knowledge (6 items).
- Treatment experience (13 items).

Participants responded to statements using 5-point Likert scales (1 = no agreement to 5 = full agreement), providing information about their understanding, attitudes, and experiences with PAP therapy. Demographic items collected contextual information including age, gender, OSA disease duration (defined as the time interval between self-reported diagnosis of OSA and date of study participation), and treatment history (PAP or non-PAP therapies, received at the center or at external institutions), to enable subgroup and comparative analyses.

### Statistical analysis

Only fully completed questionnaires were included in the analysis, resulting in a complete-case dataset with no missing values across analyzed variables. Data analysis was conducted using SPSS software (Version 26.0, IBM). Negatively phrased items (including items 4.8 and 4.10) were reverse-coded prior to analysis, so that higher values consistently represented more favorable outcomes across all measures. Statistical analysis included descriptive statistics, comparative analysis using Spearman’s rho and Kendall’s tau tests, and linear regression modeling. No sensitivity analysis was conducted, as the exploratory design did not involve varying model specifications or thresholds. Linear regression analysis was chosen given the quasi-continuous nature of Likert-scale average values. Significance levels were set at *p* <. 050 for the analysis.

## Results

### Sample characteristics

A total of 148 questionnaires with sufficient data were available for analysis. The study cohort consisted of predominantly male participants (72.8%), with a mean age of 57.9 ± 11.8 years. OSA disease history was defined as the time since patient-reported diagnosis. PAP adherence was classified based on self-reported current use versus discontinuation and verified by therapy device reports when available. Disease chronicity was notable, with most participants (59.4%) having been diagnosed with OSA for more than 36 months (Table 1). The remaining participants were distributed across shorter disease durations, with 21.8% diagnosed between 24 and 36 months prior, and 18.8% diagnosed within the previous 24 months.

**Table 1:** Sample characteristics (*N* = 148)

Variable	Value
Age (years, mean ± SD)	57.9 ± 11.8
Gender (f/m, %)	23.2/72.8
PAP usage (%)	
Adherent Non-adherent	58.8 41.2
OSA disease history (%)	
< 6 months 6–12 months 12–24 months 24–36 months > 36 months	6.0 5.3 7.5 21.8 59.4
Alternative OSA treatment (%)	
Yes No	70.9 29.1

At the time of study participation, 58.8% (*n* = 87) of participants were adherent to their prescribed PAP therapy, while 41.2% (*n* = 61) had discontinued treatment. Participants adherent to PAP therapy were significantly older than those that discontinued treatment (60.6 ± 10.5 vs. 53.6 ± 12.3 years, *p* <. 001). There was no significant difference in the gender distribution across both cohorts.

Use of multiple devices was defined as documented transitions between different PAP models or masks during the course of treatment. Most participants (61.8%) had experience with a single PAP device, while 38.2% had used multiple devices throughout their treatment course. Regarding mask interfaces, 29.6% had used a single mask type, while the majority (70.4%) had experience with multiple masks during their therapy. The number of alternative treatments reflects the average number of non-PAP therapies previously attempted per patient. Most participants (70.9%) reported no experience with therapeutic options beyond PAP therapy. Among those who had sought alternative treatments, nasal surgery was the most common (14.0%), followed by oral appliance therapy (11.2%), while other interventions such as positional therapy, tonsillectomy, uvulopalatopharyngoplasty, and tongue-base surgery were each utilized by less than 7% of participants (Table 2).

**Table 2:** Alternative treatments used in patients adherent and non-adherent to PAP therapy

Variable	PAP adherent	PAP non-adherent
Number of alternative treatments (mean ± SD)	0.10 ± 0.40	0.93 ± 0.99
Oral appliance (%)	1.2	25.0
Positional therapy (%)	0.0	15.0
Nasal surgery (%)	4.6	26.7
Tonsillectomy (%)	4.8	8.3
UPPP (%)	1.2	10.0
Tongue-base surgery (%)	1.2	10.0

### Reliability and factor structure

Internal consistency of the patient experience questionnaire was assessed using Cronbach’s alpha across the four domains (Table 3). The overall reliability varied between scales, with Cronbach’s alpha ranging from 0.677 to 0.915, indicating acceptable to excellent internal consistency. Factor analysis confirmed the underlying structure, with the Kaiser-Meyer-Olkin measure supporting sampling adequacy and Bartlett’s test confirming sufficient correlation among items (all domains *p* <. 001). Full statistical details, including item-total correlations and factor loadings, are provided in the supplementary material.

**Table 3:** Reliability and factor structure of questionnaire domains, including cronbach’s alpha for internal consistency, Kaiser-Meyer-Olkin (KMO) measure for sampling adequacy, and bartlett’s test of sphericity for factorability

Domain	Number of items	Cronbach’s alpha	KMO measure	Bartlett’s Test (χ², df, *p*-value)
Health attitudes and self-management	6	0.677	0.708	χ² = 267.6, df = 21, *p* <. 001
OSA care provision perceptions	10	0.812	0.736	χ² = 623.6, df = 66, *p* <. 001
OSA disease knowledge	6	0.733	0.663	χ² = 428.7, df = 15, *p* <. 001
Treatment experience	11	0.915	0.888	χ² = 768.5, df = 66, *p* <. 001

### Health attitudes and self-management

**Fig. 1:**
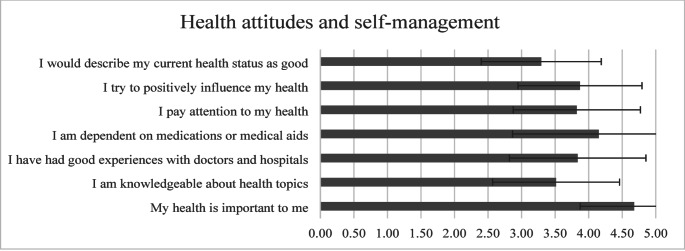
Likert scores (mean ± SD) for the domain *Health attitudes and self-management*

The average domain score was 3.82 ± 0.61. Participants strongly valued their health, with 93.8% rating “*My health is important to me*” as 4 or 5 (mean 4.68 ± 0.81). Active PAP users reported significantly higher reliance on medical aids compared to those who discontinued treatment (4.52 ± 0.98 vs. 3.64 ± 1.48, *p* <. 001). Health literacy was moderate, with 44.5% rating their health knowledge as high, representing values of 4 or 5 on the Likert scale (Fig. 1).

### OSA care provision perceptions

**Fig. 2:**
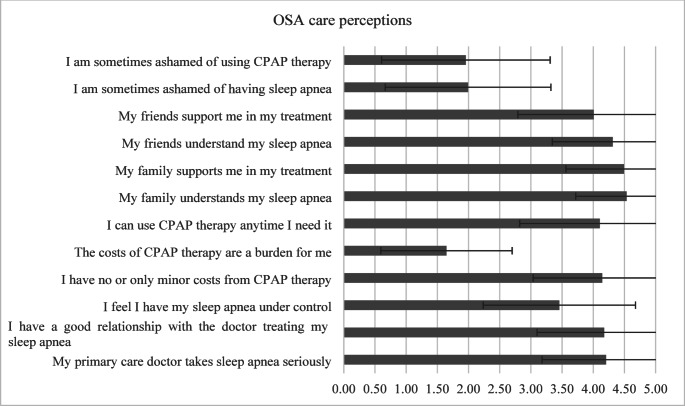
Likert scores (mean ± SD) for the domain *OSA care perceptions*

With a mean domain score of 3.83 ± 1.06, family support emerged as particularly strong. Over 90% reported high family understanding and support for their OSA treatment. Physician support was also positive, with 77.9% agreeing their primary care physician took sleep apnea seriously. Only 52.2% felt they had their sleep apnea under control, with significantly lower ratings among those who discontinued PAP therapy (*p* <. 001). Notably, over 70% reported low stigma associated with their condition or treatment (Fig. 2).

### OSA disease knowledge

**Fig. 3:**
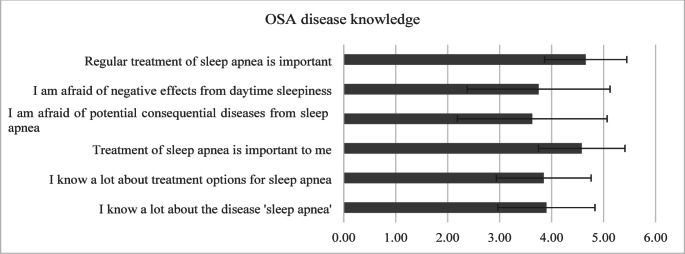
Likert scores (mean ± SD) for the domain *OSA disease knowledge*

This domain (mean 3.83 ± 1.09) revealed strong recognition of treatment importance, with over 90% acknowledging the importance of regular OSA treatment. Most participants demonstrated good disease understanding, with approximately 70% reporting high knowledge of both OSA and its treatment options. Significant concerns about OSA consequences were reported, with around 63% expressing fear of complications and 65% worried about daytime sleepiness impacts (Fig. 3).

### Treatment experience

**Fig. 4:**
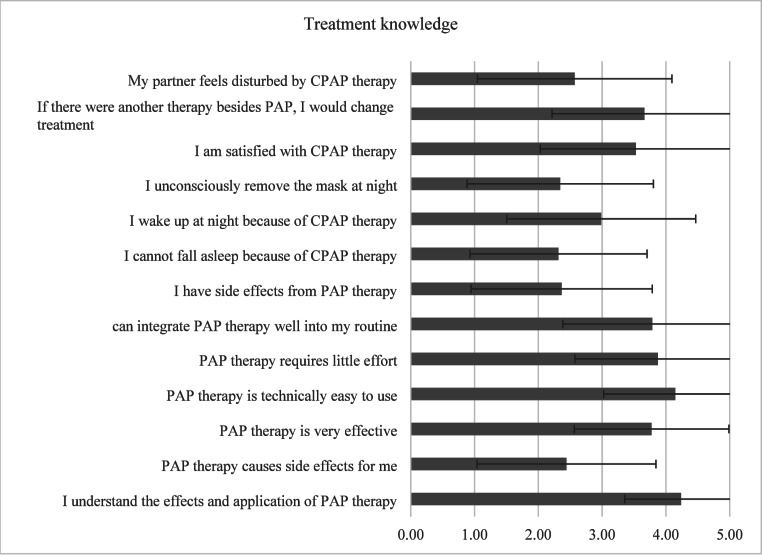
Likert scores (mean ± SD) for the domain *Treatment experience*

The mean domain score for “*Treatment experience*” was 3.23 ± 1.32. Understanding of PAP therapy mechanics was high (88.2% rated 4 or 5), and most participants (73.3%) found the therapy technically easy to use (Fig. 4). However, side effects were common, with 55.1% reporting PAP-related issues and 57.9% experiencing sleep initiation problems. Despite these challenges, only 21.1% reported reducing therapy use due to side effects, and overall satisfaction was moderate to high, with 59.1% rating their satisfaction as 4 or 5 (mean 3.53 ± 1.50).

### Predictors of patient satisfaction

Overall satisfaction with PAP therapy was moderate to high, with 59.1% rating their satisfaction as 4 or 5 (mean 3.53 ± 1.50). Patient satisfaction with PAP therapy showed significant associations with multiple factors. Satisfaction ratings negatively correlated with the use of alternative OSA treatments (τ = -0.274, *p* <. 001) and the total number of alternative treatments tried (*r* = -.270, *p* <. 001). Patients reporting higher satisfaction were less likely to pursue non-PAP alternatives. PAP satisfaction ratings also showed significant positive correlations with disease duration (τ = 0.187, *p* =. 008), duration of PAP therapy usage (τ = 0.185, *p* =. 011), and number of devices used (τ = 0.153, *p* =. 031).

The number of PAP masks tried was positively correlated with patient satisfaction, though this association did not reach statistical significance (τ = 0.130, *p* =. 063). No significant correlation was found between patient satisfaction and current PAP usage (τ = 0.113, *p* =. 115).

A hierarchical regression analysis was first conducted using domain-level scores, excluding patient satisfaction as the dependent variable, which revealed *Treatment experience* as the primary predictor of PAP therapy satisfaction (β = 0.826, 95% CI [0.720, 0.932], *p* <. 001), while other variables including *OSA disease knowledge*, *OSA care provision perceptions*, and *Health attitudes and self-management* did not significantly contribute to the model. Subsequent item-level analysis within the *Treatment experience* domain further explored the specific factors driving patient satisfaction, using stepwise multiple regression. The final model included four predictor items and was statistically significant (F(4, 88) = 69.375, *p* <. 001), explaining 75.9% of the variance in patient satisfaction (R² = 0.759, Adjusted R² = 0.748).

The items entered into the final model, in order of inclusion, were:

1. Item 4.6 „*I can integrate PAP therapy well into my routine*” (β = 0.286, 95% CI [0.142, 0.431], *p* =. 001)
2. Item 4.8 „*I wake up at night due to PAP therapy*” (reverse-coded, β = 0.273, 95% CI [0.126, 0.420], *p* <. 001)
3. Item 4.3 „*PAP therapy is effective*” (β = 0.304, 95% CI [0.130, 0.478], *p* <. 001)
4. Item 4.10 „*I unconsciously remove the mask at night*” (reverse-coded, β = 0.198, 95% CI [0.053, 0.343], *p* =. 007)

Item 4.6 was the strongest predictor, uniquely explaining 59.7% of the variance in reported patient satisfaction. The addition of item 4.8 in the second model increased the explained variance by 11% to a total of 70.7%. Items 4.3 and 4.10 in the third and fourth models further increased the explained variance by 3.1% and 2.1% respectively. The standardized beta coefficients indicate the relative importance of each predictor, with item 4.3 (β = 0.304) having the greatest impact, followed by item 4.6 (β = 0.286), item 4.8 (β = 0.273), and item 4.10 (β = 0.198).

### Temporal patterns of PAP satisfaction predictors

To examine how satisfaction predictors evolve over treatment course, participants were stratified by disease duration into early stage (< 12 months, *n* = 17), intermediate stage (12–36 months, *n* = 43), and established stage (> 36 months, *n* = 88). Separate regression analyses were conducted for each group using the previously identified predictor variables.

In the early stage, satisfaction was primarily driven by therapy effectiveness (*r* =. 696, *p* <. 010) and showed strong negative correlation with nocturnal awakening (*r*=-.885, *p* <. 001). The model explained 85.5% of satisfaction variance. For intermediate stage patients, therapy effectiveness remained important (*r* =. 695, *p* <. 001), while routine integration emerged as a significant predictor (β = 0.444, 95% CI [0.240, 0.648], *p* <. 001). This model explained 71.3% of variance.

Among established users, therapy effectiveness maintained its significance (*r* =. 764, *p* <. 001), while the impact of mask removal decreased (*r*=-.572, *p* <. 001). The final model explained 72.1% of variance, with effectiveness remaining the dominant predictor (β = 0.508, 95% CI [0.295, 0.721], *p* <. 001). All temporal models showed good fit (R² >0.70), suggesting consistent determinants of satisfaction once patients progress beyond initial adaptation.

## Discussion

This study provides new insights into factors influencing patient experience and satisfaction with PAP therapy for OSA, highlighting the complex relationship between treatment-specific aspects, patient knowledge, and care provision. As a cross-sectional study conducted at a tertiary referral center, the findings are limited by the inherent inability to infer causality and by potential selection bias. Our findings demonstrate that specific therapy experiences are stronger predictors of satisfaction than general health attitudes or care delivery perceptions. The regression model identified four key predictors explaining 75.9% of satisfaction variance, emphasizing the importance of treatment effectiveness, integration into daily routine, and management of disruptions during sleep. The significant positive association between patient satisfaction and reverse-coded items related to nocturnal awakening and mask removal indicates that patients who experience fewer sleep disruptions due to their therapy report higher overall satisfaction. These findings suggest that clinical interventions should focus not only on optimizing treatment efficacy but also on supporting patients in integrating therapy into their daily lives and their sleep routine.

The strong predictive value of treatment effectiveness aligns with recent research demonstrating that excessive daytime sleepiness is significantly associated with lower patient satisfaction with healthcare providers and overall care provision [13]. Our findings extend beyond simple treatment effectiveness by demonstrating that practical aspects of therapy integration into daily life are equally crucial. This suggests that clinicians should consider implementing structured support programs focused on helping patients develop sustainable routines around their PAP therapy, particularly in the early adaptation period.

The high importance of routine integration as a predictor (β = 0.286) suggests that successful PAP therapy requires more than just clinical efficacy. Recent research has shown that device usability significantly predicts PAP adherence among older adults, explaining 15% of variation in nightly hours of use beyond demographics and other factors [14]. The development of screening tools to identify patients who might struggle with routine integration could allow for early intervention and targeted support. This approach could be particularly valuable given that early intervention is crucial for establishing long-term adherence patterns [15].

The significant correlation between satisfaction and disease duration (τ = 0.187) suggests a potential learning curve in PAP therapy adaptation. This temporal aspect of treatment experience has been underexplored in previous research, which has typically focused on short-term adherence metrics. Healthcare providers may benefit from adjusting their support strategies based on treatment duration, offering more intensive practical support early in treatment while focusing on maintaining motivation and addressing emerging challenges in chronic use.

The negative correlation between satisfaction and alternative treatment seeking (τ=-0.274) provides new insights into treatment trajectories, suggesting that early dissatisfaction may drive the exploration of alternatives rather than vice versa. This finding has important implications for clinical practice, indicating that early identification and intervention with dissatisfied patients might prevent unnecessary treatment switching and improve overall outcomes.

Our findings regarding family support, with > 90% positive ratings, underscore the social context of chronic disease management from the patients’ perspective. Recent studies have found that partner engagement directly influences CPAP adherence, particularly when marital quality is high [16]. However, interestingly, while support systems were rated highly in our study, they did not emerge as significant predictors of satisfaction in the regression model. This suggests that while social support may be necessary, it is not sufficient for treatment satisfaction without accompanying treatment effectiveness and practical manageability. Clinicians should thus consider developing strategies to effectively engage family members while maintaining focus on practical aspects of therapy management.

It is noteworthy that women, with only 23.2% of the sample, were underrepresented in this study, a pattern consistent with prior research where OSA is often underdiagnosed in women due to sex-specific symptom presentations. Although our analysis did not stratify by gender due to limited subgroup power, previous studies have shown that women may report greater discomfort with PAP interfaces and may respond differently to support interventions [17]. Additionally, digital health tools and telemonitoring platforms have shown promise in improving PAP adherence [18, 19]. Future studies should aim to explore gender-specific drivers of satisfaction and the role of patient-focused design in improving outcomes across diverse populations.

The findings from the temporal pattern analysis suggest an evolution in factors driving PAP therapy satisfaction. Early-stage satisfaction is influenced strongly by managing nocturnal awakenings and establishing therapy effectiveness, while routine integration and mask management gain importance over time. Therapy effectiveness remains consistently important across all stages, though its relative contribution varies. The high explanatory power of models across all temporal stages (R² >0.70) suggests that while the relative importance of factors shifts, the core determinants of satisfaction remain stable after initial adaptation. This temporal pattern indicates the need for stage-specific support strategies in clinical practice, with particular attention to effectiveness and sleep disruption in early treatment, transitioning to focus on routine integration for established users. Furthermore, clustering approaches have begun to identify distinct phenotypes of PAP users based on behavioral and clinical traits [20, 21]. Integrating these frameworks with our findings may improve predictive modeling and patient stratification.

Recent developments in OSA treatment alternatives make understanding PAP therapy satisfaction increasingly relevant. Studies of patient-reported experiences with neurostimulation therapy for example, have shown high satisfaction rates and minimal impact of side effects on therapy continuation, contrasting with our findings of side effect reporting in PAP users [22]. Recent research on oral appliance therapy shows relatively high regular usage rates of 64–68% at follow-up, with adherence primarily driven by patients’ subjective comfort and perceived improvement in snoring rather than objective disease measures.

Patient-reported experience measures in our study revealed important insights about treatment barriers that might not be captured in routine clinical assessments. The high rates of self-reported side effects (55.1%) and sleep initiation problems (57.9%) align with findings from a systematic review that identified “*Discomfort from and around PAP*” as a major theme in patient experiences [23]. However, the relatively low impact of these issues on overall satisfaction suggests that patients may accept certain challenges if they perceive the treatment as effective overall. This finding supports the development of comprehensive patient education programs that address both the technical aspects of PAP therapy and strategies for managing common side effects.

The strong influence of routine integration on satisfaction suggests that early intervention focusing on practical adaptation strategies may be as important as traditional technical support. Healthcare providers might benefit from implementing structured programs that combine technical education with practical lifestyle integration strategies, particularly during the initial treatment period. This would potentially improve not only clinical efficacy, but also economic effectiveness of PAP therapy in general. Regular assessment of routine integration challenges by collecting direct feedback regularly, could help identify patients at risk for poor adherence before satisfaction and usage decline significantly. Recent work using digital PAP tracking platforms has highlighted the role of feedback and telemonitoring in improving adherence [24, 25, 26].

### Limitations

This study has several limitations that should be taken into consideration. First, the cross-sectional design cannot establish causal relationships between identified predictors and satisfaction and can thus be used for hypothesis generation only. Longitudinal studies are required to understand how satisfaction evolves over time and whether early predictors remain stable. Second, our sample was drawn from a single tertiary sleep center in Germany, which potentially limits generalizability to other healthcare contexts or patient populations. Furthermore, the relatively high proportion of long-term users in our sample, with almost 60% using PAP therapy for longer than 36 months, may have introduced survival bias, potentially overrepresenting successfully adapted patients. Also, the uneven distribution of participants across temporal groups and potential confounding factors such as age and comorbidities may limit the interpretation of the observed effects. Potential biases include recall bias, given reliance on self-reported outcomes, and selection bias due to recruitment from a tertiary sleep center. These may limit generalizability and underestimate dissatisfaction.

Additionally, the significant age difference observed between cohorts may have confounded the results, as previous research suggests that older patients often demonstrate different adherence patterns and satisfaction levels with PAP therapy compared to younger populations, potentially influencing the identified predictors of treatment satisfaction.

Moreover, given the nature of follow-up care in tertiary sleep centers, patients who are dissatisfied or non-adherent with PAP therapy may be less likely to schedule follow-up visits and thus be underrepresented in our sample. This introduces potential survival bias, which may have influenced observed satisfaction rates.

Despite these limitations, we believe that this study adds to the body of evidence on predictors of PAP satisfaction and adherence and provides relevant new information for clinicians managing patients with OSA.

## Conclusion

This study improves our understanding of patient satisfaction with PAP therapy by identifying key predictors and emphasizing the importance of practical treatment aspects alongside clinical effectiveness. The findings demonstrate that while clinical efficacy is essential, integrating therapy into daily life and sleep routine, as well as managing treatment-related disruptions are equally important for patient satisfaction.

These insights can help in developing more patient-centered approaches to OSA management by highlighting areas for targeted intervention to improve treatment outcomes with PAP therapy. By recognizing the complex relationship between disease knowledge, practical implementation, and patient experience, healthcare providers can better support individuals throughout their treatment journey.

Implementing these findings in practice may lead to the development of personalized support strategies that address both clinical and practical aspects of PAP therapy, ultimately fostering better treatment adherence and improved patient outcomes.

## Supplementary Information

Below is the link to the electronic supplementary material.

Supplementary Material 1

## Data Availability

The data that support the findings of this study are available from the corresponding author, M.B., upon reasonable request.
