# Supplementary material for: Determinants of patient satisfaction in continuous positive airway pressure therapy for obstructive sleep apnea: A multivariate analysis of knowledge, technical, and psychosocial factors
Source: Sleep Breath. 2025 Aug 7;29(4):265. doi: 10.1007/s11325-025-03438-5 (PMC12331856; doi:10.1007/s11325-025-03438-5)
Supplement: Supplementary file 1 — Supplementary Material 1 [file 11325_2025_3438_MOESM1_ESM.docx]

Electronic supplement to

**Predictors of Patient Satisfaction in Positive Airway Pressure Therapy for Obstructive Sleep Apnea: A Multivariate Analysis of Knowledge, Technical, and Psychosocial Factors**

Marcel Braun ^1,2^, Sarah Dietz-Terjung ^1,2^, Torsten Eggert ^1,2^, Christoph Schoebel MD ^1,2^

^1^ Department of Pneumology, University Medicine Essen – Ruhrlandklinik, West German Lung Center, University Duisburg-Essen, Duisburg, Germany

^2^ Faculty of Sleep and Telemedicine, University Medicine Essen – Ruhrlandklinik, West German Lung Center, University Duisburg-Essen, Duisburg, Germany

1. **Questionnaire items**

| **Domain** | **#** | **Item** |
| --- | --- | --- |
| *1 - Health attitudes and self-management* | Q1 | My health is important to me. |
|  | Q2 | I am well versed in health issues. |
|  | Q3 | I have had good experiences with doctors and clinics. |
|  | Q4 | I am dependent on medication or aids. |
|  | Q5 | I try to pay attention to my health. |
|  | Q6 | I try to have a positive influence on my health. |
|  | Q7 | I would describe my current health status as good. |
| *2 - OSA care provision perceptions* | Q1 | My primary care doctor takes sleep apnea seriously . |
|  | Q2 | I have a good relationship with the doctor treating my sleep apnea. |
|  | Q3 | I feel I have my sleep apnea under control. |
|  | Q4 | I have no or only minor costs from CPAP therapy. |
|  | Q5 | The costs of CPAP therapy are a burden for me. |
|  | Q6 | I can use CPAP therapy anytime I need it. |
|  | Q7 | My family understands my sleep apnea. |
|  | Q8 | My family supports me in my treatment. |
|  | Q9 | My friends understand my sleep apnea. |
|  | Q10 | My friends support me in my treatment. |
|  | Q11 | I am sometimes ashamed of having sleep apnea. |
|  | Q12 | I am sometimes ashamed of using CPAP therapy. |
| *3 - OSA disease knowledge* | Q1 | I know a lot about the disease 'sleep apnea' |
|  | Q2 | I know a lot about treatment options for sleep apnea |
|  | Q3 | Treatment of sleep apnea is important to me |
|  | Q4 | I am afraid of potential consequential diseases from sleep apnea |
|  | Q5 | I am afraid of negative effects from daytime sleepiness |
|  | Q6 | Regular treatment of sleep apnea is important |
| *4 - Therapy experience* | Q1 | I understand the effects and application of PAP therapy |
|  | Q2 | PAP therapy causes side effects for me |
|  | Q3 | PAP therapy is very effective |
|  | Q4 | PAP therapy is technically easy to use |
|  | Q5 | PAP therapy requires little effort |
|  | Q6 | I can integrate PAP therapy well into my routine |
|  | Q7 | I have side effects from PAP therapy |
|  | Q8 | I cannot fall asleep because of PAP therapy |
|  | Q9 | I wake up at night because of PAP therapy |
|  | Q10 | I unconsciously remove the mask at night |
|  | Q11 | I am satisfied with PAP therapy |
|  | Q12 | If there were another therapy besides PAP, I would change treatment |
|  | Q13 | My partner feels disturbed by PAP therapy |

1. **Reliability analysis**
   1. Domain 1: *Health attitudes and self-management*
      1. Reliability statistics

| **Cronbach's Alpha** | **Cronbach's Alpha Based on Standardized Items** | **N of Items** |
| --- | --- | --- |
| 0.677 | 0.709 | 7 |

- - 1. Inter-item statistics

|  | Q1.1 | Q1.2 | Q1.3 | Q1.4 | Q1.5 | Q1.6 | Q1.7 |
| --- | --- | --- | --- | --- | --- | --- | --- |
| Q1.1 | 1.000 | 0.322 | 0.471 | 0.161 | 0.519 | 0.449 | 0.141 |
| Q1.2 | 0.322 | 1.000 | 0.295 | 0.096 | 0.380 | 0.287 | 0.044 |
| Q1.3 | 0.471 | 0.295 | 1.000 | 0.103 | 0.355 | 0.235 | 0.205 |
| Q1.4 | 0.161 | 0.096 | 0.103 | 1.000 | 0.054 | 0.012 | -0.157 |
| Q1.5 | 0.519 | 0.380 | 0.355 | 0.054 | 1.000 | 0.796 | 0.289 |
| Q.16 | 0.449 | 0.287 | 0.235 | 0.012 | 0.796 | 1.000 | 0.372 |
| Q1.7 | 0.141 | 0.044 | 0.205 | -0.157 | 0.289 | 0.372 | 1.000 |

- - 1. Summary item statistics

|  | **Mean** | **Min.** | **Max.** | **Range** | **Max-Min** | **Variance** | **Items** |
| --- | --- | --- | --- | --- | --- | --- | --- |
| Inter-Item Covariances | 0.230 | -0.182 | 0.714 | 0.896 | -3.915 | 0.033 | 7 |

- - 1. Item total statistics

|  | **Scale Mean if Item Deleted** | **Scale Variance if Item Deleted** | **Corrected Item-Total Correlation** | **Squared Multiple Correlation** | **Cronbach's Alpha if Item Deleted** |
| --- | --- | --- | --- | --- | --- |
| Q1.1 | 22.54 | 12.536 | 0.578 | 0.390 | 0.598 |
| Q1.2 | 23.66 | 13.039 | 0.378 | 0.190 | 0.644 |
| Q1.3 | 23.35 | 12.410 | 0.441 | 0.287 | 0.626 |
| Q1.4 | 23.08 | 14.226 | 0.068 | 0.064 | 0.757 |
| Q1.5 | 23.38 | 11.516 | 0.662 | 0.689 | 0.565 |
| Q1.6 | 23.33 | 11.936 | 0.574 | 0.666 | 0.590 |
| Q1.7 | 23.87 | 14.457 | 0.207 | 0.194 | 0.685 |

- - 1. Domain statistics

| **Mean** | **Variance** | **Std. Deviation** | **Items** |
| --- | --- | --- | --- |
| 27.20 | 16.628 | 4.078 | 7 |

- 1. Domain 2: *OSA care provision perceptions*
     1. Reliability statistics

| **Cronbach's Alpha** | **Cronbach's Alpha Based on Standardized Items** | **N of Items** |
| --- | --- | --- |
| 0.812 | 0.829 | 10 |

- - 1. Inter-item statistics

|  | Q2.1 | Q2.2 | Q2.3 | Q2.4 | Q2.5 | Q2.6 | Q2.7 | Q2.7 | Q2.8 | Q2.8 | Q2.9 | Q2.10 |
| --- | --- | --- | --- | --- | --- | --- | --- | --- | --- | --- | --- | --- |
| Q2.1 | 1.000 | 0.324 | 0.357 | 0.079 | 0.109 | 0.360 | 0.337 | 0.422 | 0.468 | 0.503 | 0.163 | 0.250 |
| Q2.2 | 0.324 | 1.000 | 0.311 | 0.482 | 0.241 | 0.489 | 0.387 | 0.360 | 0.480 | 0.413 | -0.005 | 0.023 |
| Q2.3 | 0.357 | 0.311 | 1.000 | 0.155 | 0.033 | 0.379 | 0.250 | 0.211 | 0.331 | 0.335 | 0.214 | 0.313 |
| Q2.4 | 0.079 | 0.482 | 0.155 | 1.000 | 0.383 | 0.399 | 0.351 | 0.358 | 0.302 | 0.240 | -0.003 | 0.100 |
| Q2.5 | 0.109 | 0.241 | 0.033 | 0.383 | 1.000 | 0.147 | 0.204 | 0.260 | 0.102 | 0.105 | -0.019 | 0.047 |
| Q2.6 | 0.360 | 0.489 | 0.379 | 0.399 | 0.147 | 1.000 | 0.492 | 0.457 | 0.540 | 0.445 | 0.002 | 0.126 |
| Q2.7 | 0.337 | 0.387 | 0.250 | 0.351 | 0.204 | 0.492 | 1.000 | 0.685 | 0.820 | 0.354 | 0.016 | 0.028 |
| Q2.8 | 0.422 | 0.360 | 0.211 | 0.358 | 0.260 | 0.457 | 0.685 | 1.000 | 0.621 | 0.594 | 0.008 | 0.151 |
| Q2.9 | 0.468 | 0.480 | 0.331 | 0.302 | 0.102 | 0.540 | 0.820 | 0.621 | 1.000 | 0.579 | 0.143 | 0.088 |
| Q2.10 | 0.503 | 0.413 | 0.335 | 0.240 | 0.105 | 0.445 | 0.354 | 0.594 | 0.579 | 1.000 | 0.132 | 0.246 |
| Q2.11 | 0.163 | -0.005 | 0.214 | -0.003 | -0.019 | 0.002 | 0.016 | 0.008 | 0.143 | 0.132 | 1.000 | 0.750 |
| Q2.12 | 0.250 | 0.023 | 0.313 | 0.100 | 0.047 | 0.126 | 0.028 | 0.151 | 0.088 | 0.246 | 0.750 | 1.000 |

- - 1. Summary item statistics

|  | **Mean** | **Min.** | **Max.** | **Range** | **Max-Min** | **Variance** | **Items** |
| --- | --- | --- | --- | --- | --- | --- | --- |
| Inter-Item Covariances | 0.325 | -0.028 | 1.328 | 1.356 | -46.953 | 0.052 | 12 |

- - 1. Item total statistics

|  | **Scale Mean if Item Deleted** | **Scale Variance if Item Deleted** | **Corrected Item-Total Correlation** | **Squared Multiple Correlation** | **Cronbach's Alpha if Item Deleted** |
| --- | --- | --- | --- | --- | --- |
| Q2.1 | 45.72 | 49.848 | 0.515 | 0.378 | 0.795 |
| Q2.2 | 45.76 | 48.648 | 0.527 | 0.435 | 0.793 |
| Q2.3 | 46.53 | 48.680 | 0.448 | 0.283 | 0.800 |
| Q2.4 | 45.83 | 49.445 | 0.424 | 0.391 | 0.802 |
| Q2.5 | 45.66 | 52.564 | 0.231 | 0.200 | 0.819 |
| Q2.6 | 45.80 | 46.556 | 0.575 | 0.446 | 0.787 |
| Q2.7 | 45.44 | 50.320 | 0.576 | 0.795 | 0.793 |
| Q2.8 | 45.48 | 48.895 | 0.616 | 0.653 | 0.788 |
| Q2.9 | 45.60 | 48.652 | 0.672 | 0.814 | 0.784 |
| Q2.10 | 45.88 | 46.788 | 0.600 | 0.602 | 0.785 |
| Q2.11 | 45.92 | 51.395 | 0.235 | 0.631 | 0.823 |
| Q2.12 | 45.89 | 49.256 | 0.353 | 0.658 | 0.811 |

- - 1. Domain statistics

| **Mean** | **Variance** | **Std. Deviation** | **Items** |
| --- | --- | --- | --- |
| 49.96 | 57.632 | 7.592 | 12 |

- 1. Domain 3: *OSA disease knowledge*
     1. Reliability statistics

| **Cronbach's Alpha** | **Cronbach's Alpha Based on Standardized Items** | **N of Items** |
| --- | --- | --- |
| 0.733 | 0.777 | 6 |

- - 1. Inter-item statistics

|  | Q3.1 | Q3.2 | Q3.3 | Q3.4 | Q3.5 | Q3.6 |
| --- | --- | --- | --- | --- | --- | --- |
| Q3.1 | 1.000 | 0.755 | 0.601 | 0.126 | 0.097 | 0.585 |
| Q3.2 | 0.755 | 1.000 | 0.375 | 0.005 | -0.028 | 0.406 |
| Q3.3 | 0.601 | 0.375 | 1.000 | 0.250 | 0.226 | 0.829 |
| Q3.4 | 0.126 | 0.005 | 0.250 | 1.000 | 0.651 | 0.331 |
| Q3.5 | 0.097 | -0.028 | 0.226 | 0.651 | 1.000 | 0.307 |
| Q3.6 | 0.585 | 0.406 | 0.829 | 0.331 | 0.307 | 1.000 |

- - 1. Summary item statistics

|  | **Mean** | **Min.** | **Max.** | **Range** | **Max-Min** | **Variance** | **Items** |
| --- | --- | --- | --- | --- | --- | --- | --- |
| Inter-Item Covariances | 0.367 | -0.035 | 1.309 | 1.344 | -37.086 | 0.098 | 6 |

- - 1. Item total statistics

|  | **Scale Mean if Item Deleted** | **Scale Variance if Item Deleted** | **Corrected Item-Total Correlation** | **Squared Multiple Correlation** | **Cronbach's Alpha if Item Deleted** |
| --- | --- | --- | --- | --- | --- |
| Q3.1 | 20.43 | 13.609 | 0.534 | 0.690 | 0.682 |
| Q3.2 | 20.49 | 14.889 | 0.336 | 0.592 | 0.729 |
| Q3.3 | 19.79 | 13.591 | 0.604 | 0.716 | 0.669 |
| Q3.4 | 20.76 | 11.607 | 0.440 | 0.445 | 0.720 |
| Q3.5 | 20.63 | 12.103 | 0.415 | 0.441 | 0.724 |
| Q3.6 | 19.71 | 13.376 | 0.684 | 0.723 | 0.654 |

- - 1. Domain statistics

| **Mean** | **Variance** | **Std. Deviation** | **Items** |
| --- | --- | --- | --- |
| 24.36 | 18.040 | 4.247 | 6 |

- 1. Domain 4: *Therapy experience*
     1. Reliability statistics

| **Cronbach's Alpha** | **Cronbach's Alpha Based on Standardized Items** | **N of Items** |
| --- | --- | --- |
| 0.915 | 0.915 | 13 |

- - 1. Inter-item statistics

|  | Q4.1 | Q4.2 | Q4.3 | Q4.4 | Q4.5 | Q4.6 | Q4.7 | Q4.8 | Q4.9 | Q4.10 | Q4.11 | Q4.12 | Q4.13 |
| --- | --- | --- | --- | --- | --- | --- | --- | --- | --- | --- | --- | --- | --- |
| Q4.1 | 1.000 | 0.152 | 0.394 | 0.318 | 0.206 | 0.376 | 0.085 | 0.178 | 0.166 | 0.154 | 0.296 | 0.104 | 0.061 |
| Q4.2 | 0.152 | 1.000 | 0.488 | 0.520 | 0.364 | 0.467 | 0.725 | 0.494 | 0.480 | 0.385 | 0.481 | 0.442 | 0.360 |
| Q4.3 | 0.394 | 0.488 | 1.000 | 0.670 | 0.556 | 0.695 | 0.457 | 0.557 | 0.519 | 0.362 | 0.726 | 0.431 | 0.378 |
| Q4.4 | 0.318 | 0.520 | 0.670 | 1.000 | 0.730 | 0.803 | 0.444 | 0.579 | 0.463 | 0.487 | 0.656 | 0.393 | 0.371 |
| Q4.5 | 0.206 | 0.364 | 0.556 | 0.730 | 1.000 | 0.682 | 0.319 | 0.462 | 0.316 | 0.341 | 0.525 | 0.307 | 0.282 |
| Q4.6 | 0.376 | 0.467 | 0.695 | 0.803 | 0.682 | 1.000 | 0.368 | 0.623 | 0.543 | 0.583 | 0.737 | 0.425 | 0.480 |
| Q4.7 | 0.085 | 0.725 | 0.457 | 0.444 | 0.319 | 0.368 | 1.000 | 0.401 | 0.388 | 0.247 | 0.391 | 0.309 | 0.249 |
| Q4.8 | 0.178 | 0.494 | 0.557 | 0.579 | 0.462 | 0.623 | 0.401 | 1.000 | 0.688 | 0.654 | 0.684 | 0.529 | 0.460 |
| Q4.9 | 0.166 | 0.480 | 0.519 | 0.463 | 0.316 | 0.543 | 0.388 | 0.688 | 1.000 | 0.582 | 0.605 | 0.516 | 0.456 |
| Q4.10 | 0.154 | 0.385 | 0.362 | 0.487 | 0.341 | 0.583 | 0.247 | 0.654 | 0.582 | 1.000 | 0.626 | 0.521 | 0.486 |
| Q4.11 | 0.296 | 0.481 | 0.726 | 0.656 | 0.525 | 0.737 | 0.391 | 0.684 | 0.605 | 0.626 | 1.000 | 0.531 | 0.530 |
| Q4.12 | 0.104 | 0.442 | 0.431 | 0.393 | 0.307 | 0.425 | 0.309 | 0.529 | 0.516 | 0.521 | 0.531 | 1.000 | 0.405 |
| Q4.13 | 0.061 | 0.360 | 0.378 | 0.371 | 0.282 | 0.480 | 0.249 | 0.460 | 0.456 | 0.486 | 0.530 | 0.405 | 1.000 |

- - 1. Summary item statistics

|  | **Mean** | **Min.** | **Max.** | **Range** | **Max-Min** | **Variance** | **Items** |
| --- | --- | --- | --- | --- | --- | --- | --- |
| Inter-Item Covariances | 0.837 | 0.085 | 1.545 | 1.460 | 18.210 | 0.118 | 13 |

- - 1. Item total statistics

|  | **Scale Mean if Item Deleted** | **Scale Variance if Item Deleted** | **Corrected Item-Total Correlation** | **Squared Multiple Correlation** | **Cronbach's Alpha if Item Deleted** |
| --- | --- | --- | --- | --- | --- |
| Q4.1 | 42.05 | 147.611 | 0.276 | 0.229 | 0.920 |
| Q4.2 | 42.82 | 131.940 | 0.637 | 0.617 | 0.909 |
| Q4.3 | 42.50 | 132.954 | 0.737 | 0.679 | 0.906 |
| Q4.4 | 42.16 | 133.221 | 0.765 | 0.742 | 0.905 |
| Q4.5 | 42.46 | 134.461 | 0.589 | 0.582 | 0.911 |
| Q4.6 | 42.45 | 127.688 | 0.807 | 0.776 | 0.902 |
| Q4.7 | 42.79 | 135.395 | 0.512 | 0.556 | 0.914 |
| Q4.8 | 42.63 | 129.078 | 0.763 | 0.647 | 0.904 |
| Q4.9 | 43.29 | 129.750 | 0.687 | 0.566 | 0.907 |
| Q4.10 | 42.63 | 130.955 | 0.651 | 0.595 | 0.909 |
| Q4.11 | 42.81 | 124.367 | 0.816 | 0.727 | 0.901 |
| Q4.12 | 43.97 | 133.367 | 0.586 | 0.411 | 0.911 |
| Q4.13 | 42.90 | 133.315 | 0.537 | 0.367 | 0.914 |

- - 1. Domain statistics

| **Mean** | **Variance** | **Std. Deviation** | **Items** |
| --- | --- | --- | --- |
| 46.29 | 154.557 | 12.432 | 13 |

1. **Factor analysis**
   1. Domain 1: *Health attitudes and self-management*

| **KMO and Bartlett's Test** | | |
| --- | --- | --- |
| Kaiser-Meyer-Olkin Measure of Sampling Adequacy. | | 0.708 |
| Bartlett's Test of Sphericity | Approx. Chi-Square | 267.585 |
|  | df | 21 |
|  | Sig. | 0.000 |

| **Total Variance Explained** | | | | | | |
| --- | --- | --- | --- | --- | --- | --- |
| Component | Initial Eigenvalues | | | Rotation Sums of Squared Loadings | | |
|  | Total | % of Variance | Cumulative % | Total | % of Variance | Cumulative % |
| 1 | 2.830 | 40.433 | 40.433 | 2.693 | 38.471 | 38.471 |
| 2 | 1.242 | 17.742 | 58.174 | 1.379 | 19.703 | 58.174 |
| 3 | 0.838 | 11.969 | 70.143 |  |  |  |
| 4 | 0.809 | 11.553 | 81.696 |  |  |  |
| 5 | 0.654 | 9.344 | 91.040 |  |  |  |
| 6 | 0.442 | 6.320 | 97.361 |  |  |  |
| 7 | 0.185 | 2.639 | 100.000 |  |  |  |

| **Rotated Component Matrix^a^** | | |
| --- | --- | --- |
|  | Component | |
|  | 1 | 2 |
| Q1.1 | 0.782 |  |
| Q1.2 | 0.616 |  |
| Q1.3 | 0.645 |  |
| Q1.4 |  | -0.681 |
| Q1.5 | 0.796 |  |
| Q1.6 | 0.699 | 0.487 |
| Q1.7 |  | 0.729 |
| Extraction Method: Principal Component Analysis.   Rotation Method: Varimax with Kaiser Normalization.^a^ | | |
| ^a^ Rotation converged in 3 iterations. | | |

| **Component Transformation Matrix** | | |
| --- | --- | --- |
| Component | 1 | 2 |
| 1 | 0.956 | 0.294 |
| 2 | 0.294 | -0.956 |
| Extraction Method: Principal Component Analysis.  Rotation Method: Varimax with Kaiser Normalization. | | |

- 1. Domain 2: *OSA care provision perceptions*

| **KMO and Bartlett's Test** | | |
| --- | --- | --- |
| Kaiser-Meyer-Olkin Measure of Sampling Adequacy. | | 0.736 |
| Bartlett's Test of Sphericity | Approx. Chi-Square | 623.555 |
|  | df | 66 |
|  | Sig. | 0.000 |

| **Total Variance Explained** | | | | | | |
| --- | --- | --- | --- | --- | --- | --- |
| Component | Initial Eigenvalues | | | Rotation Sums of Squared Loadings | | |
|  | Total | % of Variance | Cumulative % | Total | % of Variance | Cumulative % |
| 1 | 4.513 | 37.609 | 37.609 | 3.946 | 32.883 | 32.883 |
| 2 | 1.870 | 15.586 | 53.195 | 1.941 | 16.172 | 49.055 |
| 3 | 1.220 | 10.167 | 63.362 | 1.717 | 14.307 | 63.362 |
| 4 | 0.926 | 7.721 | 71.083 |  |  |  |
| 5 | 0.786 | 6.551 | 77.634 |  |  |  |
| 6 | 0.643 | 5.358 | 82.992 |  |  |  |
| 7 | 0.544 | 4.531 | 87.523 |  |  |  |
| 8 | 0.479 | 3.990 | 91.512 |  |  |  |
| 9 | 0.420 | 3.498 | 95.010 |  |  |  |
| 10 | 0.318 | 2.653 | 97.663 |  |  |  |
| 11 | 0.184 | 1.536 | 99.199 |  |  |  |
| 12 | 0.096 | 0.801 | 100.000 |  |  |  |

| **Rotated Component Matrix^a^** | | | |
| --- | --- | --- | --- |
|  | Component | | |
|  | 1 | 2 | 3 |
| Q2.1 | 0.660 |  |  |
| Q2.2 | 0.541 |  | 0.476 |
| Q2.3 | 0.469 |  |  |
| Q2.4 |  |  | 0.803 |
| Q2.5 |  |  | 0.789 |
| Q2.6 | 0.674 |  |  |
| Q2.7 | 0.773 |  |  |
| Q2.8 | 0.754 |  |  |
| Q2.9 | 0.876 |  |  |
| Q2.10 | 0.733 |  |  |
| Q2.11 |  | 0.897 |  |
| Q2.12 |  | 0.923 |  |
| Extraction Method: Principal Component Analysis.  Rotation Method: Varimax with Kaiser Normalization.^a^ | | | |
| ^a^ Rotation converged in 6 iterations. | | | |

| **Component Transformation Matrix** | | | |
| --- | --- | --- | --- |
| Component | 1 | 2 | 3 |
| 1 | 0.909 | 0.203 | 0.364 |
| 2 | -0.090 | 0.948 | -0.306 |
| 3 | -0.407 | 0.245 | 0.880 |
| Extraction Method: Principal Component Analysis.  Rotation Method: Varimax with Kaiser Normalization. | | | |

- 1. Domain 3: *OSA disease knowledge*

| **KMO and Bartlett's Test** | | |
| --- | --- | --- |
| Kaiser-Meyer-Olkin Measure of Sampling Adequacy. | | 0.663 |
| Bartlett's Test of Sphericity | Approx. Chi-Square | 428.716 |
|  | df | 15 |
|  | Sig. | 0.000 |

| **Total Variance Explained** | | | | | | |
| --- | --- | --- | --- | --- | --- | --- |
| Component | Initial Eigenvalues | | | Rotation Sums of Squared Loadings | | |
|  | Total | % of Variance | Cumulative % | Total | % of Variance | Cumulative % |
| 1 | 2.966 | 49.436 | 49.436 | 2.725 | 45.409 | 45.409 |
| 2 | 1.602 | 26.704 | 76.140 | 1.844 | 30.731 | 76.140 |
| 3 | 0.715 | 11.921 | 88.061 |  |  |  |
| 4 | 0.348 | 5.800 | 93.861 |  |  |  |
| 5 | 0.215 | 3.579 | 97.440 |  |  |  |
| 6 | 0.154 | 2.560 | 100.000 |  |  |  |

| **Rotated Component Matrix^a^** | | |
| --- | --- | --- |
|  | Component | |
|  | 1 | 2 |
| Q3.1 | 0.905 |  |
| Q3.2 | 0.810 |  |
| Q3.3 | 0.792 |  |
| Q3.4 |  | 0.877 |
| Q3.5 |  | 0.884 |
| Q3.6 | 0.783 |  |
| Extraction Method: Principal Component Analysis.  Rotation Method: Varimax with Kaiser Normalization.^a^ | | |
| ^a^ Rotation converged in 3 iterations. | | |

| **Component Transformation Matrix** | | |
| --- | --- | --- |
| Component | 1 | 2 |
| 1 | 0.907 | 0.421 |
| 2 | -0.421 | 0.907 |
| Extraction Method: Principal Component Analysis.  Rotation Method: Varimax with Kaiser Normalization. | | |

- 1. Domain 4: *Therapy experience*

| **KMO and Bartlett's Test** | | |
| --- | --- | --- |
| Kaiser-Meyer-Olkin Measure of Sampling Adequacy. | | 0.888 |
| Bartlett's Test of Sphericity | Approx. Chi-Square | 786.459 |
|  | df | 66 |
|  | Sig. | 0.000 |

| **Total Variance Explained** | | | | | | | |
| --- | --- | --- | --- | --- | --- | --- | --- |
| Component | Initial Eigenvalues | | | | Rotation Sums of Squared Loadings | | |
|  | Total | % of Variance | Cumulative % | Total | | % of Variance | Cumulative % |
| 1 | 5.892 | 49.104 | 49.104 | 3.415 | | 28.454 | 28.454 |
| 2 | 1.292 | 10.764 | 59.868 | 2.821 | | 23.509 | 51.963 |
| 3 | 1.097 | 9.138 | 69.005 | 2.045 | | 17.042 | 69.005 |
| 4 | 0.827 | 6.888 | 75.893 |  | |  |  |
| 5 | 0.622 | 5.184 | 81.077 |  | |  |  |
| 6 | 0.546 | 4.546 | 85.623 |  | |  |  |
| 7 | 0.495 | 4.126 | 89.748 |  | |  |  |
| 8 | 0.300 | 2.502 | 92.251 |  | |  |  |
| 9 | 0.295 | 2.454 | 94.705 |  | |  |  |
| 10 | 0.251 | 2.095 | 96.800 |  | |  |  |
| 11 | 0.216 | 1.802 | 98.602 |  | |  |  |
| 12 | 0.168 | 1.398 | 100.000 |  | |  |  |

| **Rotated Component Matrix^a^** | | | |
| --- | --- | --- | --- |
|  | Component | | |
|  | 1 | 2 | 3 |
| Q4.1 |  | 0.714 |  |
| Q4.2 |  |  | 0.815 |
| Q4.3 |  | 0.675 |  |
| Q4.4 |  | 0.735 |  |
| Q4.5 |  | 0.714 |  |
| Q4.6 | 0.509 | 0.729 |  |
| Q4.7 |  |  | 0.910 |
| Q4.8 | 0.736 |  |  |
| Q4.9 | 0.727 |  |  |
| Q4.10 | 0.821 |  |  |
| Q4.11 | 0.691 |  |  |
| Q4.12 | 0.704 |  |  |
| Extraction Method: Principal Component Analysis.  Rotation Method: Varimax with Kaiser Normalization.^a^ | | | |
| ^a^ Rotation converged in 5 iterations. | | | |

| **Component Transformation Matrix** | | | |
| --- | --- | --- | --- |
| Component | 1 | 2 | 3 |
| 1 | 0.685 | 0.578 | 0.444 |
| 2 | -0.586 | 0.799 | -0.136 |
| 3 | -0.433 | -0.167 | 0.886 |
| Extraction Method: Principal Component Analysis.  Rotation Method: Varimax with Kaiser Normalization. | | | |

1. **Stratification of responses by PAP adherence**

**Fig. S1: Likert scores in patients PAP adherent vs. PAP non-adherent (mean ± SD) for the domain *Health attitudes and self-management* (* = p<.050)**

**Fig. S2: Likert scores in patients PAP adherent vs. PAP non-adherent (mean ± SD) for the domain *OSA care provision perceptions* (* = p<.050)**

**Fig. S3: Likert scores in patients PAP adherent vs. PAP non-adherent (mean ± SD) for the domain *OSA disease knowledge* (* = p<.050)**

**Fig. S4: Likert scores in patients PAP adherent vs. PAP non-adherent (mean ± SD) for the domain *Therapy experience* (* = p<.050)**
